# Supplementary material for: Single-photon test of hyper-complex quantum theories using a metamaterial
Source: Nat Commun. 2017 Apr 21;8:15044. doi: 10.1038/ncomms15044 (PMC5413945; doi:10.1038/ncomms15044)
Supplement: Supplementary Information — Supplementary Figures and Supplementary Notes. [file ncomms15044-s1.pdf]

Supplementary Information for Single-Photon Test of Hyper-Complex Quantum Theories Using a Metamaterial

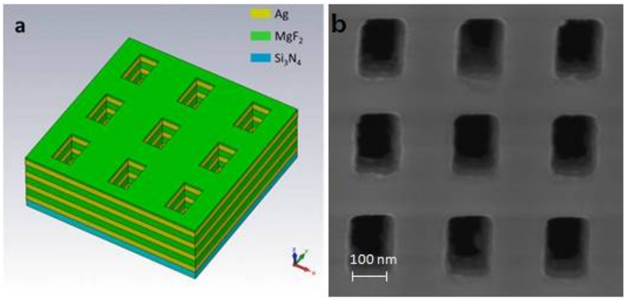

Supplementary Figure 1: Bulk negative index metamaterial. (**a**) Schematic of the silver (Ag)/ magnesium fluoride (MgF_2) multilayer fishnet metamaterials with a period of 360 nm, and a hole size of 120 nm×210 nm. Negative refractive index is obtained via the coupling between the Drude-like negative permittivity background and the multiple magnetic resonances formed between each functional layer of metal/dielectric/metal nanostructures. (**b**) SEM image of the fabricated fishnet bulk NIM structure.

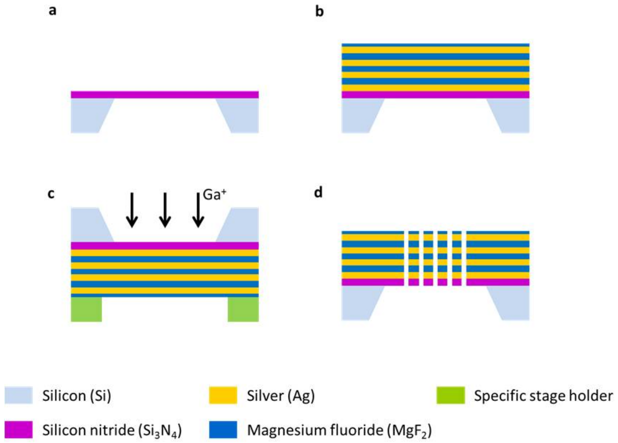

Supplementary Figure 2: Schematic of the key steps in sample fabrication. (**a**) Fabrication of an ultra-low-stress Si_3N_4 suspended membrane. (**b**) Multilayer electron beam evaporation of Ag and MgF_2 layers without vacuum break. **(c)** Flip side mounting of sample followed by Ga^+ focused ion beam milling to pattern the nanostructures. (**d**) Final fishnet structure formed.

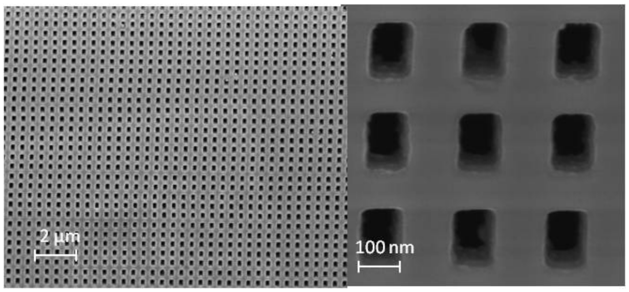

Supplementary Figure 3: SEM images of the sample. The top view showing the patterned nanostructures in periodic form, while the tilted view clearly displays the metal-dielectric multilayers.

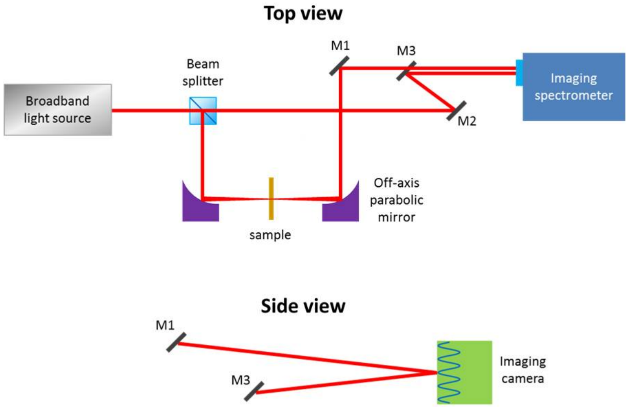

Supplementary Figure 4: Phase measurement setup. Interference between a beam transmitted through the sample and a reference beam allow the fringes to be formed at the imaging camera. By comparing the interferogram measured with and without the sample, the phase shift induced by the metamaterial can be determined simultaneously across a broad spectrum, limited only by the operational wavelength range of the broadband light source and the detector sensitivity.

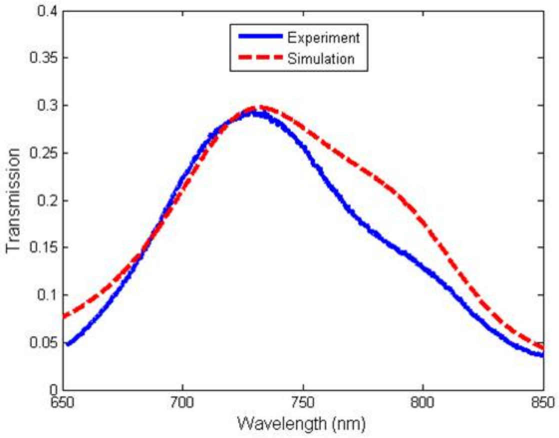

Supplementary Figure 5: Transmission of the fishnet metamaterial. Both the measured and simulated results show a broad spectrum with relatively high transmission. Maximum transmission of 30% is observed at 740 nm, while 15% transmission is measured at the desired 790 nm wavelength.

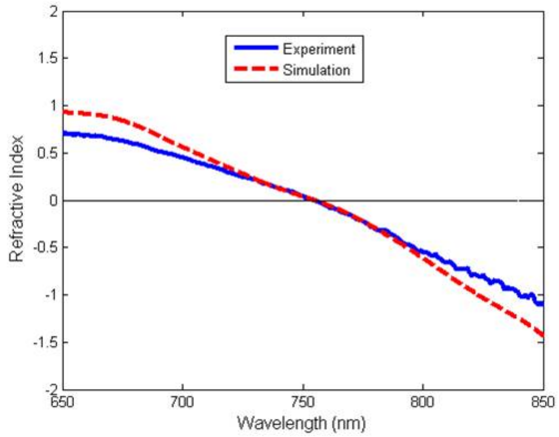

Supplementary Figure 6: Refractive index of the bulk fishnet metamaterial. Both the measured and simulated refractive index values show a gradual transition from positive to negative index, with zero-crossing at around 750 nm wavelength, and an index of -0.4 at 790 nm wavelength

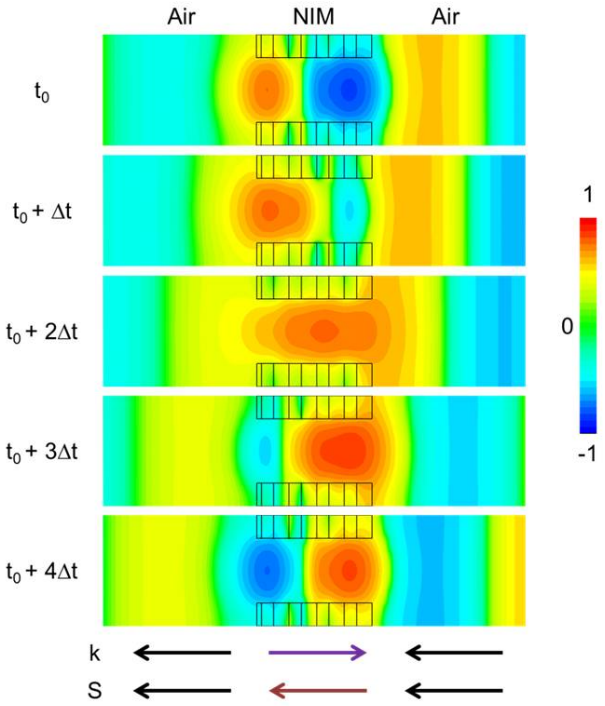

Supplementary Figure 7: Time evolution of the phase front at 790 nm. While the Poynting vector S is always conserved, the wave vector k is shown to be anti-parallel inside the negative index metamaterial. Such backward propagating waves behavior is unique for a material with negative refractive index and negative phase accumulation.

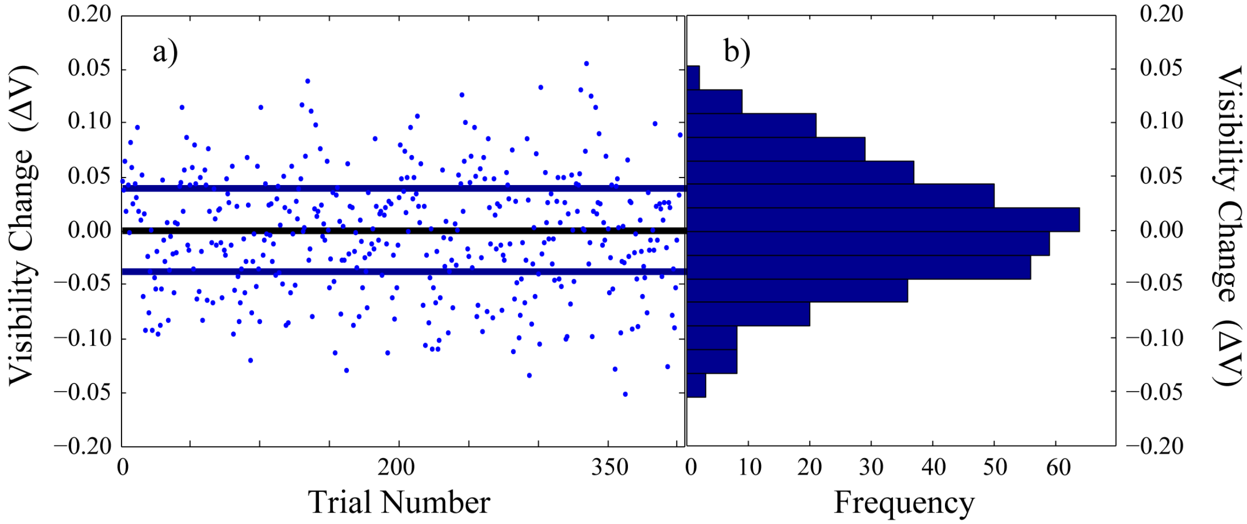

Supplementary Figure 8: Results for repeated runs of the control experiment. For these data, the negative index metamaterial was not inserted in the Sagnac interferometer. a) In this plot, each point corresponds to one run of the control experiment, consisting of turning the liquid crystal off and measuring the visibility of the Mach-Zehnder interferometer, followed by turning the liquid crystal on and remeasuring the visibility. The difference between these two visibilities for each run is plotted here for data from each of the two ports of the Mach-Zehnder interferometer. A total of 201 experimental runs were made, resulting in 402 values of ΔV. The black line marks the mean of all of the points (0.002), and the blue lines the standard deviation (0.0556). (**b**) A histogram of the data plotted in panel a).

Supplementary Note 1. Fishnet optical metamaterial

In our experiment, we used a fishnet optical metamaterial which have 7 physical layers of silver (Ag, 40 nm) and magnesium fluoride (MgF_2, 50 nm), with a 15 nm capping layer of MgF_2 to realize such a structure, see Supplementary Figure 1. The nanofabrication processes include multiple steps of electron beam evaporation of Ag and MgF_2 materials on a 50 nm-thin low stress silicon nitride membrane, followed by Gallium-based focused ion beam lithography from the membrane side. The scanning electron microscope (SEM) image of the fabricated sample is shown in Supplementary Figure 1b. To verify the negative phase response of the NIM, we used a spectrally and spatially resolved interferometry setup to characterize the sample.

Fabrication — In order to attain negative phase for light passing through the sample, a suspended fishnet negative index metamaterial (NIM) is fabricated, hence avoiding any positive phase contribution from the substrate. The fabrication of the NIM starts with a suspended 50 nm ultra-low-stress silicon nitride (Si_3N_4) membrane made from standard MEMS fabrication technologies. The metal-dielectric stack is then deposited onto the Si_3N_4 membrane using layer-by-layer electron beam evaporation technique at pressure $\approx 1 \times 10^{-6}$ Torr. The exact sequence of evaporation is three repetition of alternating silver (Ag, 40 nm) and magnesium fluoride (MgF_2, 50 nm) layers, followed by a 15 nm of MgF_2 as the capping layer to prevent oxidation from the top side. Next, the sample is turned upside down and mounted on a special stage holder which has a matching trench at the center. The nanostructures are milled by using focused ion-beam (FIB) from the membrane side. This is essential not just for alignment purpose, but also to reduce the optical loss caused by Ga ion penetration into the metal layers. The key fabrication steps are illustrated in Supplementary Figure 2. The final structure made has a slight sidewall angle along the thickness direction (Supplementary Figure 3), but is previously found to have only minor influence on the negative index property.

Supplementary Note 2. Experimental characterization setup for metamaterial

To measure the transmission phase change induced by the fishnet metamaterial across a broad frequency range, we built a spectrally and spatially resolved Mach-Zehnder inter-

ferometry setup. Essentially a broadband light source is split into two paths, one passing through the sample while the other serves as a reference beam, before recombining them at the input of an imaging spectrometer. The two beams interfere at different angles to produce interference fringes along the vertical axis of the imaging camera. A change in the optical path length of one of the beams will therefore cause the interference fringe to shift vertically on the image plane. By measuring the interferogram with and without the sample, and comparing them using Fourier analysis, the metamaterial induced phase change can therefore be obtained. Importantly, the phase change at different wavelengths can be captured simultaneously along the horizontal axis of the camera in a single-shot measurement.

Supplementary Note 3. Metamaterial design and experimental measurement

Transmission — The transmission property of the sample is important for the statistical reliability of the single photon measurement result. We performed three-dimensional full-wave finite-difference time-domain (FDTD) numerical simulations to optimize the design of the fishnet metamaterials such that the structure can attain a relatively high transmission while acquiring a negative refractive index at the desired wavelength of 790 nm. The computation is carried out using realistic material properties taking into account the dissipative loss of the silver metal used. The polarization is chosen to be E_x (see Supplementary Figure 1), which allows excitation of the anti-symmetric magnetic resonance whereby negative permeability (thus negative index) can be attained. As shown in Supplementary Figure 5, several transmission peaks are observed. This multiple resonance feature arises due to the stacking of the metal/dielectric/metal layers, which lead to a low-loss broadband transmission feature at the desired wavelength range. Also shown is the experimental measurement result which matches well with the numerical design values. In particular, the experimental transmission at 790 nm is measured to be 15%, which is among the highest reported in the visible wavelengths for bulk NIM, and is sufficient for single photon experiment.

To extract the materials effective refractive index, we simulate the transmission and reflection (both amplitude and phase) of the designed fishnet metamaterial and then reconstruct the refractive index by using the Fresnel equation. We aim for the index zero-crossing at 750 nm, above which the index will become negative. Supplementary Figure 6 shows the simulated refractive index from 650 nm to 850 nm wavelength range, essentially a smooth

transition from positive to negative index. Experimentally, using the phase shift values measured by the setup in Supplementary Figure 4, with the standard assumption of negligible multiple reflection within the structure, we could further extract its refractive index values across a broad frequency range, as depicted by the blue line in Supplementary Figure 6. The trend basically agrees with the simulated results, with the slight discrepancy most likely due to the small sidewall angle of the actual fishnet structures and other fabrication-induced errors. A broadband negative index property is thus obtained from 750 nm up to 850 nm. At the wavelength of interest 790 nm, both the designed and measured refractive index is found to be -0.4.

Negative phase — To illustrate the negative phase delay of the fishnet negative index metamaterial (NIM), we show in Supplementary Figure 7 the time evolution of the propagating phase fronts inside the bulk metamaterial at 790 nm wavelength, with the cross section taken at the center of the fishnet holes. The color map represents the normalized electric field in the x-direction. S and k are the Poynting vector and the wave vector, respectively. Unlike conventional positive refractive index metamaterials, the Poynting vector and wave vector are essentially antiparallel inside the NIM, demonstrating the negative phase accumulation and backward wave propagation behavior.
